# Aerobic exercise, an effective intervention for cognitive impairment after ischemic stroke

**DOI:** 10.3389/fnagi.2025.1514271

**Published:** 2025-04-04

**Authors:** Mingjin Zhu, Wenjun Chen, Jie Zhang

**Affiliations:** ^1^Department of Rehabilitation Medicine, Tongde Hospital of Zhejiang Province, Hangzhou, China; ^2^Department of Pharmacy, Xixi Hospital of Hangzhou, Hangzhou, China

**Keywords:** aerobic exercise, ischemic stroke, cognitive dysfunction, neuroplasticity, rehabilitation

## Abstract

Cognitive dysfunction is a common and debilitating complication following ischemic stroke, significantly impairing the quality of life of patients. In recent years, aerobic exercise has emerged as a promising non-pharmacological intervention to mitigate post-stroke cognitive impairment (PSCI). This review synthesizes current evidence on the efficacy and mechanisms of aerobic exercise in enhancing cognitive recovery after ischemic stroke. Key mechanisms include improved cerebral hemodynamics through enhanced cerebral blood flow (CBF), promotion of neuroplasticity via brain-derived neurotrophic factor (BDNF)-mediated pathways, suppression of neuroinflammation (e.g., NLRP3 inflammasome inhibition), and attenuation of oxidative stress. Preclinical and clinical studies demonstrate that aerobic exercise modalities such as gait training, cycling, and aquatic therapy enhance cognitive domains including memory, executive function, and attention, with optimal benefits observed at moderate-to-high intensity and a frequency of ≥3 sessions per week. Despite robust evidence, challenges remain in standardizing exercise protocols and addressing individual variability in treatment response. Future research should prioritize large-scale randomized controlled trials to validate long-term cognitive benefits and identify biomarkers for personalized rehabilitation strategies. This review underscores the imperative to integrate aerobic exercise into post-stroke rehabilitation paradigms, offering a dual therapeutic approach to improve both physical and cognitive outcomes.

## 1 Introduction

Ischemic stroke is a significant global health concern, characterized by the interruption of blood supply to the brain, leading to various neurological deficits. Its epidemiology reveals a high prevalence, particularly in populations with risk factors such as old age, hypertension, diabetes, and obesity (Wang et al., 2022). Reports indicate that stroke is a leading cause of long-term disability, with cognitive impairment being one of the most common sequelae following an ischemic event (Rochemont et al., 2020). Age-related physiological changes—such as hemodynamic dysfunction, loss of elasticity in the arterial walls, chronic neuroinflammation, and increased blood-brain barrier permeability—amplify vascular cognitive impairment severity (Iadecola et al., 2019). Post-stroke cognitive impairment is defined as a decline in cognitive function following a stroke, manifesting as difficulties in attention, memory, executive function, and visual-spatial abilities (Gray et al., 2021). This impairment profoundly affects the quality of life and can complicate rehabilitation efforts, emphasizing the need for effective interventions (Gallucci et al., 2024). The spectrum of cognitive impairment can range from mild cognitive dysfunction to more severe forms, such as vascular dementia (Inoue et al., 2023). Understanding the clinical presentation of these cognitive deficits is crucial for developing targeted rehabilitation strategies that can improve outcomes for stroke survivors.

Physical exercise, as a non-pharmacological intervention, can modulate neuroinflammatory pathways, enhance cognitive function, and improve brain health in Alzheimer's disease (Hu et al., 2024). Aerobic exercise, in particular, has emerged as a vital component in the rehabilitation of stroke patients, especially in addressing cognitive deficits, and plays a key role in improving recovery outcomes (Maeneja et al., 2023). Aerobic exercise, defined as physical activity that elevates heart rate and enhances cardiovascular fitness, includes activities such as walking, cycling, and swimming (Serra et al., 2022). Aerobic exercise has emerged as a promising intervention for mild cognitive impairment (MCI), while also enhancing cerebral blood flow, promoting neurogenesis, and improving cognitive function through molecular mechanisms such as modulating microglia and astrocytes, and promoting neuroprotective factors (Huang et al., 2023; Dao et al., 2024). Research indicates that engaging in regular aerobic exercise can mitigate the cognitive decline associated with ischemic stroke, making it a promising intervention for enhancing cognitive recovery and functional independence in affected individuals (Choi et al., 2024). A network meta-analysis of 29 RCTs with 1,507 participants found that aerobic exercise was the most effective for improving global cognition (MD = 2.83, 95% CI 0.66–4.85) in older adults with Alzheimer's disease (Hu et al., 2025). Another clinical study showed that one-year moderate-to-high intensity aerobic exercise intervention in sedentary older adults increased peak VO_2_ by 10% (*p* < 0.001), improved cognitive function, and showed a positive correlation between fitness gains and cognitive performance (*r* = 0.282, *p* = 0.042) (Tarumi et al., 2022).

Currently, the heterogeneity in aerobic exercise responses based on stroke subtype is unclear, and the mechanisms underlying the effects of aerobic exercise on cognitive recovery post-stroke remain insufficiently explored. This highlights a significant gap in stroke rehabilitation research, where a one-size-fits-all approach to aerobic exercise may not be universally effective. Different stroke subtypes can lead to distinct neurological impairments, which may influence the brain's response to physical activity. Additionally, the optimal exercise intensity, duration, and frequency for recovery may vary depending on the stroke subtype and individual patient characteristics. A deeper understanding of the relationship between ischemic stroke, cognitive dysfunction, and aerobic exercise is essential. Further investigation is needed to clarify how aerobic exercise improves cognitive outcomes in stroke survivors. This review integrates preclinical and clinical evidence, bridging fields such as epidemiology and molecular biology, to explore the potential mechanisms through which aerobic exercise improves cognitive impairment after ischemic stroke. The aim is to transform aerobic exercise from a general intervention into a precise tool for cognitive rehabilitation post-stroke.

## 2 The relationship between ischemic stroke and cognitive dysfunction

### 2.1 Pathophysiological mechanisms of PSCI

Ischemic stroke leads to a cascade of pathological events that can significantly impair cognitive function. The initial event typically involves the occlusion of a cerebral artery, resulting in reduced blood flow and subsequent neuronal injury due to oxygen and glucose deprivation. This ischemic insult triggers a series of neuroinflammatory responses, which exacerbate neuronal damage. For instance, the overexpression of pro-inflammatory cytokines and the activation of microglia can lead to further neuronal injury and cognitive decline (Chen et al., 2023).

The disruption of the blood-brain barrier during ischemic events allows for the infiltration of immune cells and exacerbates neuroinflammation, contributing to cognitive deficits (Zhao et al., 2022). Furthermore, studies indicate that oxidative stress plays a crucial role in the pathophysiology of stroke-related cognitive dysfunction, promoting neuronal apoptosis and impairing neurogenesis in regions such as the hippocampus (Xia et al., 2023). The interplay between these mechanisms underscores the complexity of cognitive impairment following ischemic stroke, highlighting the need for targeted therapeutic strategies that address these multifaceted processes (Li et al., 2024). Additionally, the cognitive consequences of ischemic stroke can vary significantly depending on the lesion location of the brain. Left hemispheric stroke, white matter and medial temporal lobe leision are associated with the development of neurocognitive disorder (Schellhorn et al., 2021). Another research showed that stroke in the left middle cerebral artery territory and thalamus were significantly associated with cognitive impairment, and domain-specific impairments were related to various infarct patterns across both hemispheres including the left medial temporal lobe (verbal memory) and the right parietal lobe (visuospatial functioning) (Weaver et al., 2021). Studies indicate that patients with subcortical infarcts (e.g., basal ganglia) show slower cognitive recovery but still benefit from aerobic exercise through neuroplasticity in contralateral regions (Li et al., 2022). In contrast, cortical infarcts (e.g., prefrontal cortex) may require longer intervention periods to observe cognitive improvements (Gallucci et al., 2024).

### 2.2 Types and assessment methods of cognitive dysfunction

Cognitive dysfunction following ischemic stroke can manifest in various forms, including memory deficits, executive dysfunction, and attention impairments. The assessment of these cognitive domains is crucial for understanding the extent of impairment and guiding rehabilitation efforts. Standardized neuropsychological tests, such as the Mini-Mental State Examination (MMSE) and the Montreal Cognitive Assessment (MoCA), are commonly employed to evaluate cognitive function in stroke patients (Suda et al., 2020). Cognitive impairment severity is classified as mild with a MoCA score between 18 and 25, moderate with a score between 10 and 17, and severe with a score below 10 (Suda et al., 2020). Additionally, more advanced methodologies, such as functional near-infrared spectroscopy, have been explored to provide insights into cognitive deficits by measuring brain activity in real-time (Nakamura et al., 2021). The identification of specific cognitive deficits can aid in tailoring rehabilitation strategies, as different cognitive domains may respond differently to various interventions. A comprehensive approach to cognitive assessment is essential for optimizing recovery in post-stroke patients and enhancing their overall quality of life.

## 3 Aerobic exercise interventions for stroke: different modalities

### 3.1 Types of exercise

Aerobic exercise modalities play a crucial role in rehabilitation following a stroke, with various forms such as gait training, aquatic therapy, and cycling being particularly effective (Table 1). Gait training focuses on improving walking abilities, which is vital for regaining independence post-stroke. Studies have shown that gait training can enhance lower limb strength and coordination, leading to improved mobility and reduced fall risk in stroke survivors (Meng et al., 2022). Meng's study was a randomized controlled trial (RCT) with 120 participants, and gait training (3x/week for 12 weeks) improved walking speed by 0.3 m/s (95% CI: 0.2–0.4) and reduced fall risk by 40% compared to controls. Aquatic therapy provides a low-impact alternative that minimizes joint stress while improving cardiovascular fitness, mood, and overall quality of life. An RCT with 26 participants (*n* = 26) demonstrated that aquatic therapy resulted in significant improvements in pain, resilience, and both physical and social function in stroke patients (Pérez-de la Cruz, 2020). Cycling, whether on stationary bikes or recumbent cycles, has been shown to enhance cardiovascular endurance and lower limb strength, making it a versatile option for stroke rehabilitation, as demonstrated by an 8-week cycling program (3x/week) that increased lower limb strength by 25% and 6-min walk distance by 50 meters in chronic stroke survivors (Linder et al., 2021). Gait Training enhances visuospatial/executive function through complex motor-cognitive integration (Bergqvist et al., 2023). Aquatic therapy demonstrates significant advantages in improving the emotional state of stroke patients and health-related quality of life. This may indirectly support cognitive recovery by alleviating psychological stress, but it is unclear which specific cognitive domains are improved (Veldema and Jansen, 2021). A clinical trial with 15 stroke patients demonstrated that dual-task cycling enhanced attentional control by prioritizing task focus (*p* < 0.05), alongside increased motor cortex desynchronization, reflecting strengthened motor-cognitive synergy during rehabilitation (Wang et al., 2023). Each of these modalities can be tailored to individual capabilities and preferences, thereby maximizing participation and adherence to exercise programs.

**Table 1:** Comparative efficacy of aerobic exercise modalities in stroke rehabilitation.

**Modality**	**Study design**	**Sample size**	**Intervention parameters**	**Key findings**	**Cognitive domains improved**
Gait training	RCT (Bergqvist et al., 2023)	45	3×/week, 6 weeks, dose-matched intensity	↑Walking speed by 0.3 m/s; ↑MoCA Vis/Ex score explained 34% variance in 6MWT (*p =* 0.017)	Executive function, Visuospatial ability
Aquatic therapy	RCT (Pérez-de la Cruz, 2020)	41	2×/week, 8 weeks, low intensity	↓Pain (VAS: 6.25 → 3.00, *p* < 0.001); ↑ Resilience (CD-RISC: 18.20 → 24.80, *p* < 0.001); SF-36 (physical function: 17.07 → 23.20, *p* < 0.001 and social function: 5.72 → 6.20, *p =* 0.017).	Emotional regulation, social function
Cycling	Clinical trial (Wang et al., 2023)	15	dual-task training, training period not mentioned	↑Attentional control (priority-following: *p* < 0.05); Increased motor cortex desynchronization	Attention, motor-cognitive synergy

RCT, randomized controlled trial; 6MWT, 6-min walk test; MoCA Vis/Ex, Montreal cognitive assessment—Visuospatial/Executive; VAS, visual analog scale; CD-RISC, Connor-Davidson resilience scale; SF-36, 36-item short form health survey.

### 3.2 Exercise frequency

The frequency of aerobic exercise is a critical factor in optimizing recovery and improving functional outcomes after a stroke. Current guidelines recommend that stroke survivors engage in aerobic exercise at least 3 times per week for a minimum of 8 weeks (Mackay-Lyons et al., 2020). This frequency is essential for promoting cardiovascular health and enhancing neuroplasticity, which is vital for motor recovery. The intensity of aerobic exercise is another critical consideration in stroke rehabilitation. Research indicates that high-intensity interval training (HIIT) can yield superior benefits compared to moderate-intensity continuous training (MICT) regarding motor cortex plasticity and functional recovery (Andrews et al., 2020). HIIT consists of alternating between 3 min of cycling at 50% HRR and 2 min of cycling at up to 90% HRR, for a total duration of 20 min. Moderate Intensity consists of 20 min of cycling at 50% HRR. HIIT has been shown to improve cardiovascular fitness and metabolic health more effectively, while also enhancing cognitive function and mood (Guo et al., 2022). A study compared the acute effects of HIIT (15s at 100% peak power, 15s rest, 2 × 20 min) and MICT (34 min at 60% peak power) on executive function in 25 older adults, with cognitive tests administered immediately and 45 min post-training. HIIT significantly reduced Switching reaction times more than MICT (*p* = 0.019), indicating a greater positive effect on executive functions (Ahmadi et al., 2025). However, the choice between moderate and high intensity should be individualized based on the stroke survivor's health status, functional capacity, and personal preferences. Moderate-intensity exercise is often more feasible for individuals with significant impairments, providing a safe and effective means to improve overall fitness and well-being (Tian et al., 2021).

Ultimately, a tailored approach that considers both intensity and individual capabilities is essential for maximizing rehabilitation outcomes.

## 4 Effects of aerobic exercise on cerebral blood flow

### 4.1 Correlation between cerebral blood flow and cognitive function

The relationship between CBF and cognitive function has garnered significant attention in recent research (Table 2). Studies have shown that CBF plays a critical role in maintaining cognitive performance, as it directly influences neuronal activity and metabolic processes in the brain. Specifically, a reduction in overshoot systolic cerebral blood velocity in the posterior cerebral arteries (*p* = 0.021) significantly predicted better memory performance (Monteiro et al., 2024). In particular, regions of the brain those are responsible for executive functions, such as the prefrontal cortex, exhibit a strong correlation between CBF and cognitive performance (Pellegrini-Laplagne et al., 2023). Enhanced CBF in these areas has been associated with improvements in tasks requiring attention, working memory, and decision-making (Loprinzi et al., 2020). A clinical research including 16 participants showed that aerobic exercise significantly increased cerebral blood velocity (BV) from baseline (62.82 ± 10.68 cm/s) to steady state (70.27 ± 12.63 cm/s; *p* < 0.001, d*z* = −0.95), with the BV elevation correlating strongly with improved executive function (antisaccade reaction time reduction: *r* = 0.53, *p* = 0.04), demonstrating that cerebrovascular hemodynamic enhancement (*p* < 0.05) mechanistically contributes to post-exercise cognitive improvement (Tari et al., 2020). Moreover, a 12-week aerobic exercise intervention improved CBF and cognitive function in both cognitively normal older adults and those with MCI. The exercise reversed baseline CBF differences and enhanced working memory and verbal fluency, with CBF changes linked to cognitive improvements, particularly in individuals with MCI (Alfini et al., 2019). Additionally, the findings from a systematic review indicated that aerobic exercise not only improves CBF but also positively influences cognitive flexibility and memory, further supporting the idea that maintaining healthy cerebral hemodynamics is critical for cognitive health (Haeger et al., 2019). Thus, the evidence suggests a robust relationship between aerobic exercise-induced improvements in CBF and enhanced cognitive function, highlighting the importance of physical activity in promoting brain health across the lifespan.

**Table 2:** Effects of aerobic exercise on CBF and cognitive function.

**Study**	**Design**	**Sample**	**Intervention**	**CBF measurement**	**Cognitive assessment**	**Key findings**
Monteiro et al. (2024)	Cross-sectional observational study	60 hypertension patients	NA	TCD	RAVLT, Stroop test, the trail making test parts A and B, Digit Symbol Search tests	↑ CBF in posterior arteries predicted better memory (*p =* 0.021)
Tari et al. (2020)	Crossover	16 healthy adults	Single-bout cycling	TCD, NIRS	Executive function (antisaccade task)	↑ CBF velocity (62.82 → 70.27 cm/s, *p* < 0.05); correlation with attention (*r =* 0.53, *p =* 0.04)
Alfini et al. (2019)	Longitudinal	35 older adults (17 MCI)	12-week walking (4×/week, moderate intensity)	MRI perfusion	RAVLT, Clock Drawing, Animal Fluency, COWAT	Reversed CBF deficits in MCI (*p* < 0.05)

CBF, cerebral blood flow; NA, not available; MCI, mild cognitive impairment; TCD, transcranial Doppler ultrasound; NIRS, near-infrared spectroscopy; RAVLT, Rey auditory verbal learning test; COWAT, Controlled Oral Word Association Test.

### 4.2 How aerobic exercise improves cerebral hemodynamics

Aerobic exercise has been shown to significantly enhance CBF dynamics, which is crucial for maintaining optimal brain health. Several studies have highlighted the physiological mechanisms through which aerobic exercise induces these improvements. Aerobic training can enhance endothelial function and increase nitric oxide availability, leading to improved vasodilation and increased blood flow to the brain (Tomoto and Zhang, 2024). Additionally, clinical studies indicate that although different types of exercise training do not have a significant impact on cerebrovascular reactivity in adults, aerobic training has a certain effect on resting cerebral hemodynamics (Corkery et al., 2021).

Moreover, acute bouts of aerobic exercise have been found to produce immediate increases in CBF, which may be attributed to elevated cardiac output and enhanced oxygen delivery to the brain (Mulser and Moreau, 2023). This effect is particularly pronounced in individuals with pre-existing cardiovascular conditions, who may experience greater improvements in CBF following aerobic interventions (Moncion et al., 2022). Furthermore, long-term aerobic exercise training results in sustained improvements in resting CBF, particularly in populations at risk for cognitive decline, such as older adults and those with MCI (Alfini et al., 2019). Overall, these findings underscore the critical role of aerobic exercise in enhancing cerebral hemodynamics, potentially offering protective benefits against age-related cognitive decline and neurodegenerative diseases.

## 5 The promoting effect of aerobic exercise on neuroplasticity

### 5.1 Neuroplasticity and its importance in rehabilitation

Neuroplasticity refers to the brain's ability to reorganize itself by forming new neural connections throughout life. This capacity is crucial for recovery from brain injuries and neurological disorders, as it facilitates the brain's adaptation to new situations or changes in the environment. In rehabilitation, neuroplasticity plays a vital role in restoring function and improving outcomes for patients with stroke, traumatic brain injury, and other conditions that affect the central nervous system (Montero-Almagro et al., 2024). The process of neuroplasticity involves various mechanisms, including synaptic plasticity, neurogenesis, and the recruitment of alternative neural pathways to compensate for damaged areas (Toricelli et al., 2021). Enhanced neuroplasticity can lead to improved motor skills, cognitive function, and even emotional regulation, making it a key target in therapeutic strategies aimed at rehabilitation. Systematic reviews show that interventions promoting neuroplasticity, such as physical activity and cognitive training, can significantly enhance recovery trajectories in clinical populations, underscoring the need for integrating these strategies into rehabilitation programs (Mellow et al., 2020; Penna et al., 2021).

### 5.2 Aerobic exercise promotes neuronal growth and repair

Aerobic exercise enhances neuroplasticity by promoting the release of brain-derived neurotrophic factor (BDNF), a key mediator of synaptic plasticity and neurogenesis (Oyovwi et al., 2025). Mechanistically, BDNF binds to its high-affinity receptor, tropomyosin receptor kinase B (TrkB), triggering downstream signaling pathways such as the MAPK/ERK and PI3K/Akt cascades. These pathways facilitate neuronal survival, dendritic arborization, and synaptic strengthening (Kowiański et al., 2018). For instance, preclinical studies have demonstrated that treadmill exercise increases ERK1/2 phosphorylation in the hippocampus (Alkadhi and Dao, 2019). Additionally, treadmill exercise in rodent models of stroke results in a fourfold increase in hippocampal BDNF expression (*p* < 0.05), which enhances synaptic plasticity and neurogenesis (Sayyah et al., 2022). Clinically, 30 min of aerobic exercise at a moderate intensity, but not at a mild intensity, increases serum BDNF levels in chronic post-stroke phase (Morais et al., 2018). A meta-analysis showed that moderate-intensity aerobic exercise has been associated with improvements in the structural and functional connectivity of the brain, particularly in individuals recovering from strokes (Li et al., 2022). For instance, another RCT with 33 stroke patients highlights that aerobic exercise can enhance the neuroplasticity of the contralesional hemisphere, facilitating motor recovery by promoting the recruitment of alternative neural pathways (Hill et al., 2023). Additionally, aerobic exercise has been linked to improved cognitive function and mood, further supporting its role in enhancing neuroplasticity and rehabilitation outcomes (Sampedro-Piquero and Moreno-Fernández, 2021; Abuleil et al., 2022). Overall, the integration of aerobic exercise into rehabilitation protocols offers a promising approach to enhancing neuroplasticity, thereby improving recovery and quality of life for individuals with neurological impairments.

## 6 Regulation of inflammatory response by aerobic exercise

### 6.1 Mechanisms of post-stroke inflammatory response and modulation by exercise

Following a stroke, the brain initiates a complex inflammatory cascade that profoundly impacts recovery. Central to this process is the activation of the NLRP3 inflammasome, which triggers the release of pro-inflammatory cytokines (e.g., IL-1β, IL-18) and induces pyroptosis, amplifying neuronal injury and hindering repair (Han et al., 2023b). Post-stroke neuroinflammation is further exacerbated by the polarization of microglia toward a pro-inflammatory M1 phenotype, characterized by the production of TNF-α and reactive oxygen species (ROS), which drive secondary brain damage (Shui et al., 2024). Preclinical studies highlight the therapeutic potential of aerobic exercise in modulating this response. In rodent stroke models, 10 days of treadmill training reduced NLRP3 expression by 50% and suppressed inflammasome-mediated pyroptosis by shifting microglial polarization toward the neuroprotective M2 phenotype (Liu et al., 2022). This phenotypic shift is mediated through the IL-4/JAK1-STAT6 pathway, which enhances anti-inflammatory signaling, reduces cerebral ischemia-reperfusion injury, and improves neurobehavioral outcomes (Lu et al., 2021). While initial microglial activation is protective, chronic inflammation exacerbates blood-brain barrier disruption and neuronal loss, underscoring the need for timely intervention (Candelario-Jalil et al., 2022).

### 6.2 Anti-inflammatory and cognitive protective effects of aerobic exercise

Aerobic exercise exerts systemic and brain-specific anti-inflammatory effects, crucial for mitigating post-stroke cognitive impairment. Clinically, regular exercise reduces circulating inflammatory markers such as C-reactive protein (CRP) and IL-6, attenuating neuroinflammation and its detrimental effects on synaptic plasticity (Chen et al., 2020; Feng et al., 2023). Mechanistically, exercise enhances brain-derived neurotrophic factor (BDNF) levels, which are often suppressed by inflammation, thereby promoting neurogenesis and synaptic remodeling (De Assis and Murawska-Ciałowicz, 2024).

In populations at risk for cognitive decline (e.g., older adults, mild cognitive impairment), aerobic exercise improves memory, executive function, and processing speed (Ahn and Kim, 2023). These benefits are linked to exercise-induced suppression of NLRP3 activation, reduced M1 microglial activity, and increased BDNF-mediated neuroprotection (Han et al., 2023a). Exercise-induced increases in brain-derived neurotrophic factor (BDNF) further counteract inflammation-mediated suppression of neurogenesis and synaptic remodeling, directly supporting memory, executive function, and processing speed. These dual mechanisms—suppression of NLRP3-driven neuroinflammation and enhancement of BDNF-mediated neuroplasticity—highlight the critical role of aerobic exercise in post-stroke rehabilitation.

## 7 Neuroprotective mechanisms of aerobic exercise for PSCI

Aerobic exercise has been increasingly recognized for its neuroprotective effects, particularly in the context of neurodegenerative diseases and cognitive decline. The mechanisms underlying these benefits are multifaceted, involving both biochemical and physiological changes that promote brain health. This section delves into two significant aspects of aerobic exercise's neuroprotective mechanisms: its impact on oxidative stress and its relationship with neurotrophic factors.

### 7.1 Impact of aerobic exercise on oxidative stress

Oxidative stress, characterized by an imbalance between ROS production and antioxidant defenses, is a key factor in the pathogenesis of various neurological disorders (Hassan et al., 2022). Aerobic exercise has been shown to mitigate oxidative stress through several mechanisms. For instance, regular aerobic activity enhances the body's antioxidant capacity, leading to reduced levels of oxidative markers in the plasma (El Assar et al., 2022). A clinical study demonstrated that aerobic exercise significantly decreased oxidative stress biomarkers in healthy young adults, indicating an adaptive response to exercise that bolsters the antioxidant defense system (Ammar et al., 2020). Preclinical studies demonstrate that aerobic exercise promotes mitochondrial biogenesis, which is crucial for efficient energy metabolism and ROS management in neurons (Zhang et al., 2020). This is particularly relevant in conditions such as stroke and neurodegeneration, where mitochondrial dysfunction contributes to neuronal injury (Andrabi et al., 2020). Exercise activates peroxisome proliferator-activated receptor gamma coactivator 1-alpha (PGC-1α), a master regulator of mitochondrial biogenesis. This promotes electron transport chain efficiency, reduces ROS leakage, and enhances ATP production (de Oliveira Bristot et al., 2019). A study highlights the importance of exercise training in regulating mitochondrial function, including ADP-stimulated respiration, ROS emission, and network structure, in skeletal muscle during aging, with PGC-1α playing a key role (Halling et al., 2019). Additionally, exercise-induced upregulation of antioxidant enzymes, such as superoxide dismutase (SOD) and glutathione peroxidase (GPx), along with the suppression of ROS through Nrf2/ARE pathway activation, helps alleviate oxidative stress and protect against cellular damage (Souza et al., 2022). By reducing oxidative stress, aerobic exercise not only protects neuronal integrity but also supports cognitive function, highlighting its potential as a therapeutic intervention for preserving brain health.

### 7.2 Relationship between aerobic exercise and neurotrophic factors

Neurotrophic factors, such as Brain-Derived Neurotrophic Factor (BDNF), play a crucial role in neuronal survival, growth, and plasticity. An animal study shows that aerobic exercise has been linked to increased levels of these neurotrophic factors, which are essential for maintaining cognitive function and promoting neurogenesis (Sadri et al., 2024). Research suggests that aerobic training, particularly high-intensity exercise (80% of VO_2_max), can significantly increase BDNF levels in animal models—more than five times higher than in the control group, indicating that physical activity may enhance neuroplasticity and cognitive resilience (Lee et al., 2023). However, a 12-week clinical trial involving moderate-intensity aerobic exercise found that it did not increase serum BDNF levels post-stroke (*P* = 0.1) (Maguire et al., 2023). A systematic review by Ashcroft (*n* = 687) demonstrated that high intensity aerobic exercise leads to significant improvements in BDNF levels in patients with MCI, with a pooled mean difference of 3.42 ng/mL (95% CI: 1.92–4.92) (Ashcroft et al., 2022). Additionally, the relationship between aerobic exercise and other neurotrophic factors, such as Insulin-like Growth Factor 1 (IGF-1), has been explored, showing that exercise-induced increases in these factors can facilitate recovery from neurological insults (King et al., 2019). Overall, the modulation of neurotrophic factors by aerobic exercise underscores its potential to foster a supportive environment for neuronal health and cognitive function, making it a valuable component in strategies aimed at preventing or treating neurodegenerative diseases.

**Figure 1:**
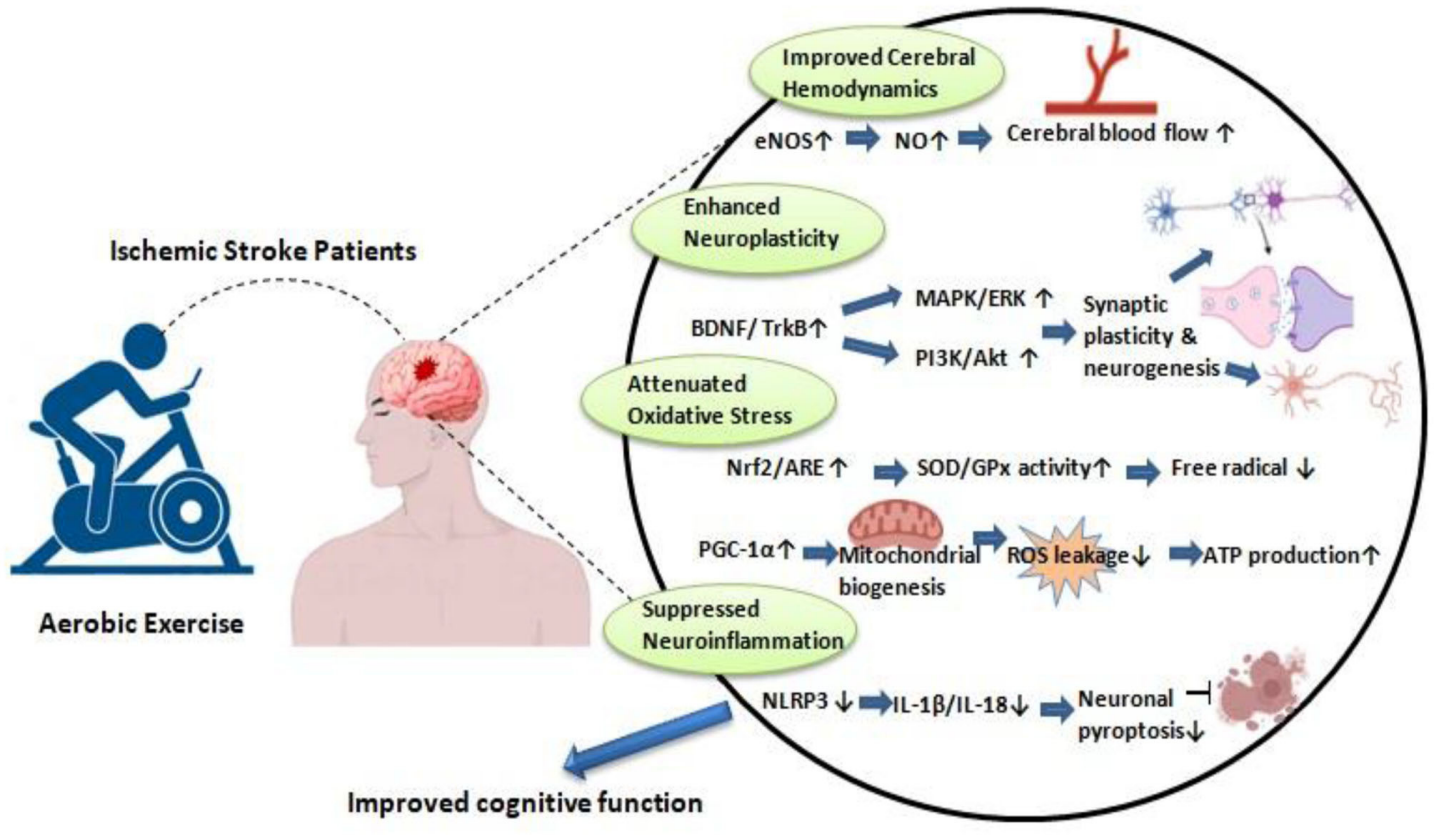
The mechanisms of aerobic exercise in mitigating PSCI.

The integrated mechanisms through which aerobic exercise ameliorates PSCI are illustrated in Figure 1.

## 8 Conclusions

Aerobic exercise is a potent non-pharmacological intervention for post-stroke cognitive recovery, exerting benefits through synergistic mechanisms: enhanced cerebral perfusion, BDNF-driven neuroplasticity and NLRP3 inflammasome suppression. This review advances a precision rehabilitation framework by mapping exercise modalities to stroke subtypes—for instance, combining aerobic-cognitive training to activate frontal networks in cortical infarcts, while targeting subcortical lesions with high-intensity gait protocols to drive contralateral plasticity. Such stratification addresses pathological heterogeneity, a critical gap in current one-size-fits-all approaches. These processes synergistically promote cognitive recovery by restoring neuronal connectivity, reducing oxidative stress, and counteracting chronic inflammation.

Despite proven efficacy, challenges remain in optimizing exercise parameters (e.g., intensity, duration) and sustaining long-term cognitive gains. These challenges are particularly prominent in elderly stroke survivors, who often face unique physical and cognitive limitations. Future research should prioritize age-stratified analyses to delineate optimal aerobic exercise parameters for elderly stroke patients. Such efforts will help identify the most effective exercise parameters for this population, enabling healthcare providers to design personalized rehabilitation strategies that optimize cognitive and physical recovery. Furthermore, exploring the underlying mechanisms in more depth will enhance our understanding of how aerobic exercise influences cognitive outcomes and may lead to the identification of key biomarkers that can predict individual responses. As healthcare systems strive to implement cost-effective strategies to enhance patient outcomes, aerobic exercise emerges as a promising approach that not only aids in physical rehabilitation but also supports cognitive health. This dual benefit reinforces the need for a shift in rehabilitation paradigms, prioritizing aerobic exercise as a fundamental component of post-stroke recovery programs. Collaborative efforts among researchers, clinicians, and policymakers are essential to translate mechanistic insights into scalable rehabilitation programs, ultimately improving functional independence and quality of life for stroke survivors.
